# Intra-aortic thrombus formation in a patient supported by Impella combined with veno-arterial extracorporeal membrane oxygenation

**DOI:** 10.1093/ehjcr/ytaf178

**Published:** 2025-04-16

**Authors:** Hidetomo Nomi, Yasushi Ueki, Koichiro Kuwahara

**Affiliations:** Department of Cardiovascular Medicine, Shinshu University School of Medicine, 3-1-1 Asahi, Matsumoto 390-8621, Japan; Department of Cardiovascular Medicine, Shinshu University School of Medicine, 3-1-1 Asahi, Matsumoto 390-8621, Japan; Department of Cardiovascular Medicine, Shinshu University School of Medicine, 3-1-1 Asahi, Matsumoto 390-8621, Japan

A 76-year-old female diagnosed with fulminant myocarditis was transferred to our hospital due to cardiogenic shock. After haemodynamic collapse, mechanical circulatory support devices including peripheral veno-arterial extracorporeal membrane oxygenation (VA-ECMO) and micro-axial pump (Impella CP, Abiomed) were initiated and the haemodynamic status was stabilized. The activated partial thromboplastin time and activated clotting time were maintained ∼60 (two times the control value) and 180 s with intravenous unfractionated heparin, respectively. Transthoracic echocardiography was consecutively performed to monitor the cardiac function; however, thrombus formation in the descending aorta was not confirmed. On Day 6, transoesophageal echocardiography to assess the position of Impella revealed a large thrombus (arrowheads) extending along the catheter shaft of the Impella CP (arrows) in the descending aorta (*Panels A* and *B*, Supplementary material online, *Videos 1* and *2*). Spontaneous echo contrast around the thrombus suggested the competition of the antegrade flow from the Impella CP with the retrograde flow from the VA-ECMO (i.e. mixing zone) (Supplementary material online, *Video 3*). Contrast-enhanced CT showed a comprehensive visualization of the thrombus (*Panels C* and *D*). On Day 18, the Impella CP was successfully explanted without thrombo-embolic events, with surgical protection of the bilateral femoral artery and percutaneous thrombectomy on the iliac artery (Supplementary material online, *Video 4*).

**Figure:**
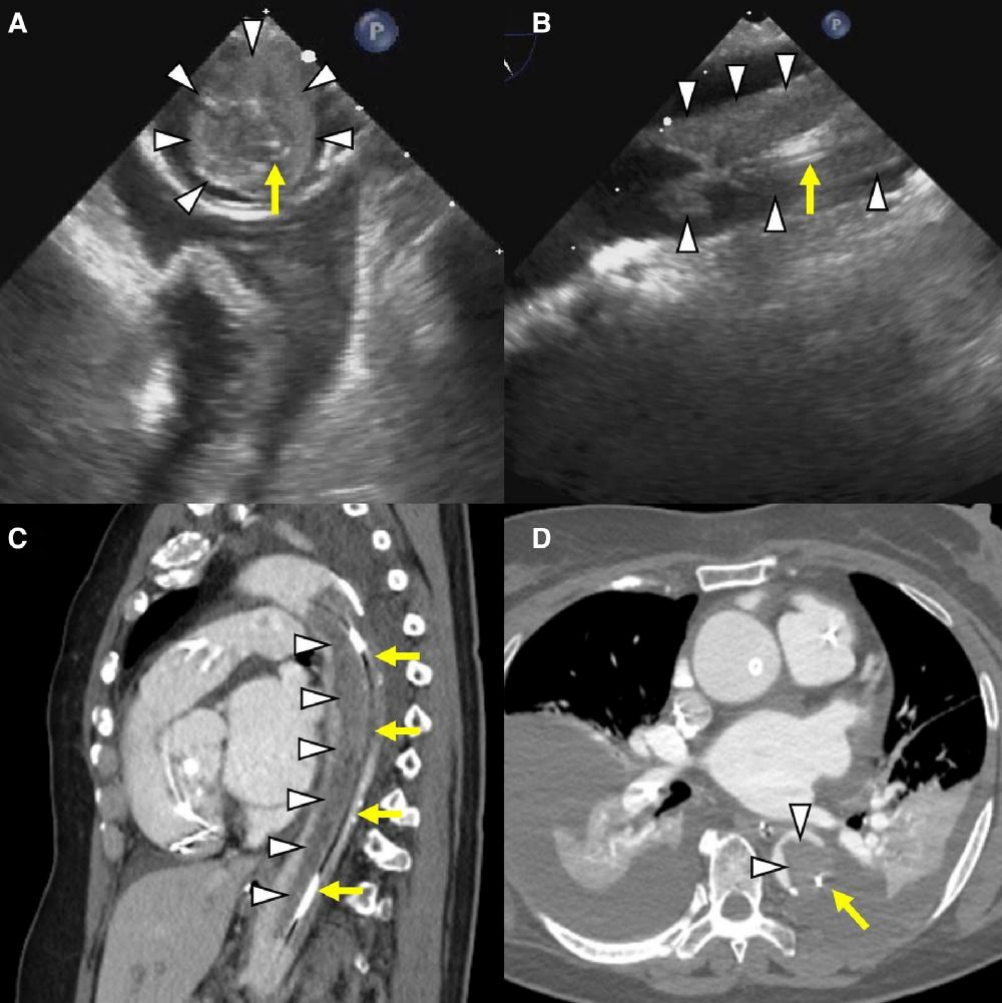


Veno-arterial extracorporeal membrane oxygenation combined with Impella (i.e. ECPELLA) is a widely-used mechanical circulatory support system for cardiogenic shock due to severe biventricular heart failure. Intra-aortic thrombus formation during the ECPELLA support is a rare but serious complication. The blood flow stagnation in the mixing zone may at least partly explain the thrombus formation despite adequate anticoagulation. This case illustrates the importance of screening thrombus formation by imaging modalities and anticoagulation management in patients supported by ECPELLA.

## Supplementary material

Supplementary material is available at *European Heart Journal – Case Reports* online.

##  

**Consent:** The patient consented to the publication of this case report in compliance with the COPE guidelines.

**Funding:** None declared.

## Supplementary Material

ytaf178_Supplementary_Data

## Data Availability

Non-identifiable data underlying this article will be made available upon reasonable request to the corresponding author.

